# Freezing of Gait can persist after an acute levodopa challenge in Parkinson’s disease

**DOI:** 10.1038/s41531-019-0099-z

**Published:** 2019-11-22

**Authors:** J. Lucas McKay, Felicia C. Goldstein, Barbara Sommerfeld, Douglas Bernhard, Sahyli Perez Parra, Stewart A. Factor

**Affiliations:** 1 Wallace H. Coulter Department of Biomedical Engineering, Georgia Tech and Emory University, Atlanta, GA USA; 2 Neuropsychology Program, Department of Neurology, Emory University, Atlanta, GA USA; 3 Jean & Paul Amos PD & Movement Disorders Program Department of Neurology, Emory University, Atlanta, GA USA

**Keywords:** Parkinson's disease, Parkinson's disease

## Abstract

Study objectives included testing whether presumed levodopa-unresponsive freezing of gait (FOG) in Parkinson’s disease (PD) actually persists in the presence of adequate dopaminergic dosing and to investigate whether the presence of other parkinsonian features and their responsiveness to therapy varies across patients without FOG (NO-FOG), with levodopa-responsive FOG (OFF-FOG), and with levodopa-unresponsive FOG (ONOFF-FOG). Fifty-five PD patients completed levodopa challenges after >12-h OFF with supratherapeutic doses of dopaminergic medications. Observed responses in FOG, measured with MDS-UPDRS-III during the patient reported full “ON”, were used to classify them as NO-FOG, OFF-FOG, or ONOFF-FOG. Serum levodopa levels were measured. Only those with ≥20% improvement in MDS-UPDRS-III score were included in analyses. Levodopa challenge was sufficient to bring about a full “ON” state with ≥20% improvement in 45 patients. Levodopa-equivalent-dose utilized was 142 ± 56% of patients’ typical morning doses. Overall, 19/45 patients exhibited FOG in the full “ON” state (ONOFF-FOG), 11 were classified as OFF-FOG, and 15 NO-FOG. Linear mixed models revealed a highly significant association between serum levodopa level and total MDS-UPDRS-III score that was similar across groups. The ONOFF-FOG group exhibited significantly higher New-FOG-questionnaire and MDS-UPDRS-II scores compared to the OFF-FOG group. Among MDS-UPDRS-III subdomains significant effects of group (highest in ONOFF-FOG) were identified for other axial parkinsonian features. We found that FOG can persist in the full “ON” state brought about by ample dopaminergic dosing in PD. Other axial measures can also be levodopa-unresponsive among those with ONOFF-FOG only. These data provide evidence that ONOFF-FOG is distinct from responsive freezing.

## Introduction

Freezing of gait (FOG), described as brief arrests of stepping when initiating gait, turning, and walking straight ahead, is a common, poorly understood symptom complex that has potentially grave consequences for Parkinson’s disease (PD) patients.^1,2^ It is unpredictable in character, is a leading cause of falls and consequent injuries, results in loss of independence and social isolation, and treatment thus far is limited.^1,3^ There has been great variability in findings related to physiological, imaging, motor, and non-motor correlates as well as therapeutic response to various treatment modalities.^4^ Recognition that FOG is not a single uniform symptom but instead exists in several subtypes may be a crucial step to improved understanding of the pathophysiology and development of new therapies.^5^

Clinical subtypes have been a subject of discussion. The phenomenology of FOG differs between patients, and may include shuffling with small steps, trembling in place without forward movement, or total akinesia.^6^ In addition, the settings such as starting, turning, or walking and the effect of special constraints (such as going through doorways) also differs between patients.^7^ It remains an open question as to whether these phenomenologies are the result of different levels of severity, different pathophysiologies, or other causes.

One other possible classification scheme may relate to pharmacology – particularly, the levodopa responsiveness of FOG. The relationship between FOG and levodopa response is complex. It is suggested through clinical experience that several apparent subtypes of levodopa responsiveness exist, including: (1) FOG which appears only in the “OFF” state, and disappears in the levodopa induced “ON” state (OFF-FOG); (2) FOG that is unresponsive and is present in the “OFF” state and persists in the “ON” state (ONOFF-FOG); and (3) FOG that is absent during the “OFF” state and present during “ON” state only (drug-induced or ON-FOG).^7–11^ The terminology used here has been previously utilized.^8^ One study indicated, based on patient questionnaire data, that 62% of FOG patients are OFF-FOG, 36% ONOFF-FOG, and 2% ON-FOG.^8^

However, the existence of levodopa-responsive subtypes is controversial. It has been argued that the “ON” state FOG in those with ONOFF-FOG represents inadequate treatment of FOG, so called Pseudo-ON FOG.^5,6,8,9^ What is certain is that the relationship of FOG to levodopa therapy has been inadequately examined.

We have previously hypothesized that ONOFF-FOG exists as a distinct form that is not the result of inadequate therapy.^10^ Here, to test this explicitly, we examined the nature of levodopa responsiveness of FOG and other parkinsonian signs in PD patients using a levodopa challenge paradigm. We examined patients in the practically defined “OFF” state and after a levodopa equivalent dose greater than their typical morning dose of medications. We measured serum levodopa levels to demonstrate that remaining FOG was not a consequence of inadequate levodopa dosage or delayed efficacy due to poor gut absorption. Our expected outcome, in addition to the persistence of FOG in the full “ON” state, was that patients with ONOFF-FOG would exhibit responses in overall parkinsonian symptoms, as assessed with MDS-UPDRS-III,^11^ to changes in serum levodopa level that were comparable to those observed in patients without FOG (NO-FOG) and with OFF-FOG. We also comprehensively investigated whether the response of individual parkinsonian symptoms to medication state varied across FOG groups.

## Results

### Levodopa challenge test

**Fig. 1:**
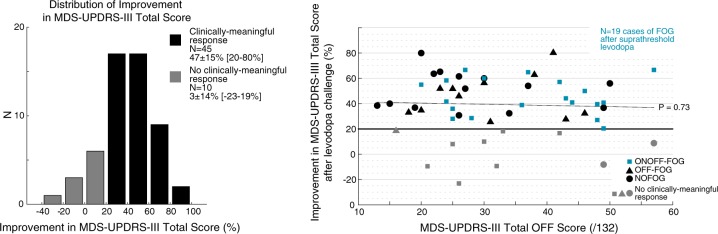
Improvement in MDS-UPDRS-III total scores after acute levodopa challenge. **a** Distribution of reduction in MDS-UPDRS-III total scores among patients who did and who did not exhibit a clinically meaningful response (black and gray bars, respectively). **b** Scatterplot depicting association between reduction in MDS-UPDRS-III total score after levodopa challenge and OFF medication score. Patients classified as ONOFF-FOG are shown in blue. Best fit trendline for entire sample is shown for reference (dashed).

Of *N* = 55 patients enrolled, *N* = 45 (82%) exhibited a full ‘ON” state plus a clinically meaningful response to the levodopa challenge (≥20% decrease MDS-UPDRS-III; Fig. 1a). The average improvement in total MDS-UPDRS-III score was 47 ± 15% (range, 20–80%) among patients who exhibited a clinically-meaningful response and 3 ± 14% (−23–19%) among patients who did not. Dyskinesia was reported in 76% of responding patients in the “ON” state (as indicated by the investigator while completing the MDS-UPDRS-III). No clear associations between improvement in MDS-UPDRS-III score or study group and baseline score were apparent on inspection of plots of improvement vs. OFF score (Fig. 1b). The average administered LED was 395 ± 243 mg (range, 133–1348 mg) in the levodopa challenge, corresponding to 142 ± 56% (range, 62–325%) of patients’ typical morning dose. Medications given during the challenge included all medications typically taken in the morning as well as additional carbidopa/levodopa. Challenge doses included: carbidopa/levodopa, in either typical or extended release formulations (all patients), ropinirole, in either typical or extended release formulations (*N* = 8), pramipexole (*N* = 4), rotigotine (*N* = 2), selegiline (*N* = 1), rasagiline (*N* = 6), entacapone (*N* = 15), and amantadine (*N* = 10). Administered doses did not significantly differ among those patients who did or did not exhibit a clinically meaningful response (levodopa-equivalent-dose in mg, *t*_53_ = 0.25, *P* = 0.81; LED as proportion of morning dose, *t*_53_ = 1.08, *P* = 0.29). Serum levodopa levels after levodopa administration were significantly higher (79%, *t*_51_ = 3.17, *P* < 0.01) among patients who did not exhibit a clinically meaningful response.

### Demographic and clinical characteristics of 3 groups

Demographic and clinical characteristics of patients who exhibited a clinically meaningful response to the levodopa challenge are summarized in Table 1. Based on changes in the MDS-UPDRS-III FOG item 11 before and after levodopa, of *N* = 45 patients who exhibited a clinically meaningful response, 19/45 (42%) were classified as ONOFF-FOG, followed by NO-FOG (15/45, 33%), and OFF-FOG (11/45, 25%). One patient initially entered as having no FOG by history had FOG on examination in the OFF state and was reclassified as OFF-FOG. Patients classified as ONOFF-FOG appeared across almost the total range of symptom levels and response levels (Fig. 1b, blue squares). None of the patients exhibited levodopa-induced ON-FOG.^9^ No significant differences between groups were observed in age, sex, education, MoCA score, age at PD onset or FOG onset, FOG duration, presence of dyskinesia during the “ON” state or MDS-UPDRS-I or IV subscores.

**Table 1:** Demographic and clinical characteristics of the study sample, overall and stratified on FOG status.

Characteristic	All *N* = 45	NO-FOG *N* = 15	OFF-FOG *N* = 11	ONOFF-FOG *N* = 19
Age (y)	66 ± 8	65 ± 11	68 ± 4	67 ± 7
Sex				
Male (*n*,%)	33 (73)	9 (60)	9 (82)	15 (79)
Female (*n*,%)	12 (27)	6 (40)	2 (18)	4 (21)
Education (y)	16 ± 2	17 ± 1	17 ± 3	16 ± 2
MoCA (/30)	25.4 ± 4.0^a^	27.4 ± 1.9	25.1 ± 4.2	24.0 ± 4.7
PD duration (y)^b^	10 ± 6	6 ± 4^c^	11 ± 5	12 ± 7^c^
Age at PD onset (y)	56 ± 9	58 ± 11	57 ± 6	55 ± 9
LED (mg)^d^	1294 ± 664	864 ± 300^c^	1197 ± 500	1690 ± 738^c^
MDS-UPDRS-I (/52)	11.8 ± 6.3	10.2 ± 5.1	11.5 ± 5.5	13.2 ± 7.5
MDS-UPDRS-II (/52)^b^	16.0 ± 7.7	10.7 ± 6.9^c^	17.3 ± 5.7	19.3 ± 7.3^c^
MDS-UPDRS-IV (/24)	6.3 ± 3.2	5.5 ± 3.1	6.3 ± 3.4	7.1 ± 3.3
ON-state dyskinesia				
Yes (*n*, %)	34 (76)	9 (60)	10 (91)	15 (79)
No (*n*, %)	11 (24)	6 (40)	1 (9)	4 (21)
FOG duration (y)	3 ± 3^e^		4 ± 3	3 ± 3
Age at FOG onset (y)	64 ± 7^e^		64 ± 6	64 ± 7
NFOG-Q (/28)^d^	20.1 ± 5.5^e^		16.5 ± 6.1^c^	22.1 ± 4.0^c^

*NO-FOG* no Freezing of Gait, *OFF-FOG* levodopa-responsive Freezing of Gait, *ONOFF-FOG* levodopa-unresponsive Freezing of Gait

^a^*N* = 43

^b^Overall effect of group at *P* < 0.05

^c^Significant difference between listed groups at P < 0.05

^d^Overall effect of group, *P* < 0.01

^e^*N* = 30

Significant contrasts were observed between the ONOFF-FOG and NO-FOG groups on PD duration (12 ± 7 vs. 6 ± 4 y, *F*_2,42_ = 5.11, *P* < 0.01) and daily LED (1690 ± 738 vs. 864 ± 300 mg, *F*_2,42_ = 9.05, *P* < 0.01). The ONOFF-FOG group exhibited significantly more impairment on NFOG-Q (*P* < 0.01) and MDS-UPDRS-II (Motor Aspects of Experiences of Daily Living) (*F*_2,42_ = 6.90, *P* < 0.01) compared to the OFF-FOG group.

Levodopa doses administered during the levodopa challenge did not vary across groups (*F*_2,41_ = 0.71, *P* = 0.50) (Table 2). When calculated as proportion of the typical morning dose, administered doses were higher in the OFF-FOG group compared to the ONOFF-FOG group (181% vs. 121%, *F*_2,41_ = 4.15, *P* = 0.02).

**Table 2:** Levodopa responsiveness of the study sample, overall and stratified on FOG status.

Characteristic	All *N* = 45	NO-FOG *N* = 15	OFF-FOG *N* = 11	ONOFF-FOG *N* = 19
Levodopa challenge dose				
Levodopa equivalent (mg)	391 ± 260	282 ± 133	438 ± 349	451 ± 263
Proportion of morning dose (%)*	138 ± 59	119 ± 31	181 ± 92^a^	128 ± 38^a^
Serum levodopa^b,c,d^				
OFF (ng/mg)	0.3 ± 0.4	0.1 ± 0.1	0.4 ± 0.3	0.5 ± 0.5
ON (ng/mg)	27.9 ± 16.8	19.9 ± 11.3^a^	28.0 ± 11.9	34.8 ± 20.8^a^
MDS-UPDRS-III (item 11 excluded)^c^				
OFF (/128)	30.8 ± 10.7	27.9 ± 10.9	29.4 ± 9.7	34.1 ± 10.8
ON (/128)	16.6 ± 8.1	13.9 ± 7.2	16.5 ± 8	18.7 ± 8.5
MDS-UPDRS-III item 11**^,c,d^				
OFF (/4)	1.6 ± 1.4	0 ± 0^a,e^	1.6 ± 0.7^a,c^	2.9 ± 0.9^c,e^
ON (/4)	0.7 ± 0.9	0 ± 0^a,e^	0 ± 0^a^	1.6 ± 0.6^e^

*NO-FOG* no Freezing of Gait, *OFF-FOG* Freezing of Gait in the OFF state only, *ONOFF-FOG* Freezing of Gait in the ON and OFF state

*^,^**Significant effect of group, *P < 0.05; **P < 0.01

^a,e^Significant difference between listed groups in post-hoc tests, *P* < 0.05. *P* values are adjusted for PD duration; summary statistics are unadjusted

^b^*N* = 43

^c^Significant effect of medication state (OFF vs. ON), P < 0.01

^d^Significant group × state interaction, *P* < 0.05

### Response of motor scores and change in serum levodopa levels after levodopa challenge in the 3 groups

MDS-UPDRS-III score (item III.11 excluded) varied significantly across medication states (ON vs. OFF; *F*_1,90_ = 53.17, *P* < 0.01) but did not vary across groups, indicating general levodopa responsiveness for all groups (Table 2). MDS-UPDRS-III scores decreased by 15.2 ± 7.1 points (range, 5–38) from the “OFF” to “ON” states, corresponding to a percent change of 47 ± 15% (range, 20–80%).

MDS-UPDRS-III item 11 varied significantly across states (*F*_1,90_ = 74.96, *P* < 0.01) and groups (*F*_2,90_ = 135.55, *P* < 0.01), and exhibited a highly significant state × group interaction (*F*_2,90_ = 19.22, *P* < 0.01), as expected by construction.

**Fig. 2:**
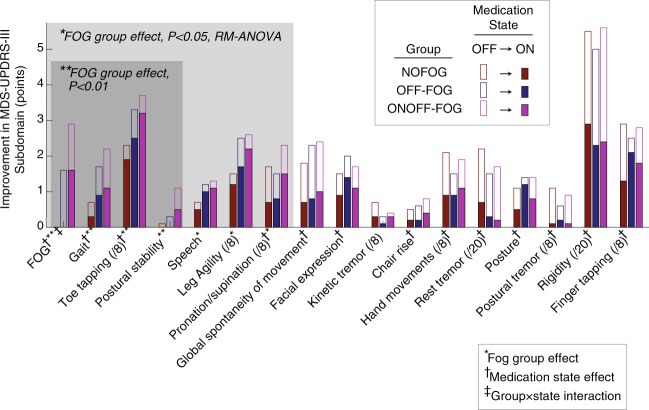
Changes in MDS-UPDRS-III subdomains from OFF to ON medication state in each study group. Empty bars indicate OFF medication scores for each subdomain; superimposed filled bars indicate ON medication scores after levodopa challenge. Study group is indicated by the colors red, blue, and purple for the NO-FOG, OFF-FOG, and ONOFF-FOG groups, respectively. Subdomains are sorted in ascending order of group effect *P* value from left to right. Maximum subdomain scores are 4 points (/4) unless noted. †Significant effect of medication state (OFF vs. ON), P < 0.05, RM-ANOVA. *,**Significant effect of group, *P < 0.05, **P < 0.01. ‡Significant state × group interaction, *P* < 0.05.

Among other MDS-UPDRS-III subdomains, significant effects of medication state were identified for all domains except for postural stability, speech, leg agility, and kinetic tremor (Fig. 2). Significant effects of group (highest among the ONOFF-FOG group) were identified for Gait (*P* < 0.01), toe tapping (*P* < 0.01), postural stability (*P* < 0.01), speech (*P* < 0.02), leg agility (*P* < 0.03), and pronation/supination of the hands (*P* < 0.05). No statistically significant state × group interactions were identified for MDS-UPDRS-III items other than FOG.

Serum levodopa level varied significantly across medication states (“ON” state 27.9 ng/mg vs. “OFF” state 0.3 ng/mg, *F*_1,86_ = 131.76, *P* < 0.01). Serum levodopa level in the “ON” state, was significantly higher in the ONOFF-FOG group compared to the NO-FOG group (34.8 vs. 19.9 ng/mg, *P* = 0.01).

### Associations between motor features and serum levodopa level in the 3 groups

**Fig. 3:**
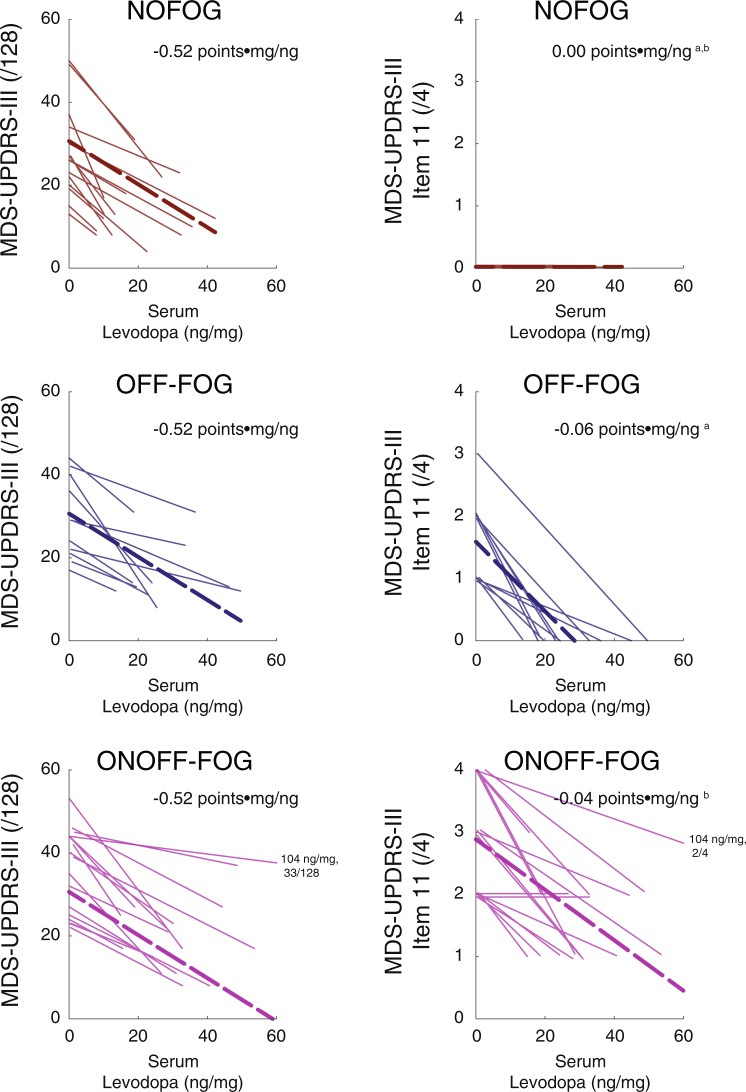
Changes in parkinsonian symptom level and changes in serum levodopa level among patients who exhibited a clinically-meaningful response to the acute levodopa challenge. Left: total MDS-UPDRS-III score (excluding item III.11, “Freezing of Gait”) and serum levodopa level. Right: MDS-UPDRS-III item III.11 and serum levodopa level. Study groups are depicted from top to bottom with colors as in Fig. 2. Thin lines depict increases in serum levodopa level and corresponding reductions in MDS-UPDRS-III from the OFF to ON states for individual patients. Scores for one patient in the ONOFF-FOG group with a very high ON state serum levodopa level (104 ng/mg) are noted. Bold dashed lines indicate linear regression fits estimated by linear mixed models. Inset: Beta values in points•mg/ng calculated by linear mixed models. ^a,b^Significant difference between slope parameters, mixed models, *P* < 0.05. Mixed model results control for PD duration. In the right panel, a small amount of vertical jitter has been added to improve visibility.

Linear mixed models revealed a highly significant association between serum levodopa level and MDS-UPDRS-III total score (item 11 excluded; *F*_1,42_ = 116.16, *P* < 0.001; Fig. 3) that did not vary across the NO-FOG, OFF-FOG, and ONOFF-FOG groups (group effect, *F*_2,47_ = 1.93, *P* = 0.16; group × serum levodopa interaction effect, *F*_2,40_ = 1.18, *P* = 0.32) indicating all groups responded in a similar manner despite the difference in FOG response. The estimated slope between MDS-UPDRS-III score and serum levodopa level was −0.52 points•mg/ng (95% CI: −0.62,−0.42). Linear mixed models revealed a highly significant group interaction effect on the association between serum levodopa level with MDS-UPDRS-III item 11 (*F*_1,41_ = 68.23, *P* < 0.001). Estimated slopes between MDS-UPDRS-III item 11 scores and serum levodopa level were 0.00 points•mg/ng (−0.02, 0.02), −0.06 points•mg/ng (−0.07, −0.04), and −0.04 points•mg/ng (−0.05, −0.03) for the NO-FOG, OFF-FOG, and ONOFF-FOG groups, respectively.

## Discussion

In this study we sought to demonstrate that FOG that is present in the “OFF” medication state can persist in some PD patients in a full “ON” state that is brought about by ample dopaminergic therapy, and therefore does not exclusively result from inadequate dopaminergic therapy (Pseudo-ON). We used rigorous methods that included subjects arriving at clinic in the practically defined “OFF” state. This was followed by a dopaminergic challenge with supratherapeutic medication doses as recommended by others.^9^ Serum levodopa levels demonstrated that “ON” FOG was not a consequence of inadequate levodopa dosage or delayed efficacy due to altered gut absorption. We required that the patient agree that they are fully “ON”, plus that they had a ≥20% response in MDS-UPDRS-III to minimize the inclusion of Pseudo-ON cases and to firmly establish responsiveness of overall parkinsonian symptoms.

Several aspects of the testing give us confidence that the levodopa challenge test was ample to bring about a full “ON” state in all patients, and especially so among those classified as ONOFF-FOG. The mean LED given in the levodopa challenge to the ONOFF-FOG group was 451 mg, 28% higher than their standard first dose. Further, 79% of these patients developed dyskinesia. The mean levodopa level was substantially higher in the ONOFF-FOG than the NO-FOG and OFF-FOG groups (Table 2). Additionally, we performed an additional ANOVA post-hoc to establish that the number of medications taken during the challenge in addition to carbidopa/levodopa was common across groups (*P* = 0.29). Taken together, these data would indicate that the FOG present in the “OFF” state is truly persistent in the “ON” state and that ONOFF-FOG is an authentic subtype.

Overall, the general presence of other cardinal parkinsonian symptoms and responsiveness of these features to levodopa also indicates that the patients we classified as ONOFF-FOG have PD and not, for example, Primary Progressive Freezing of Gait.^12^ Notably, we also did not see any cases of levodopa-induced freezing of gait (ON-FOG) in this cohort. The ONOFF-FOG subtype is more severe than the OFF-FOG subtype based on NFOGQ and is associated with more severe impairments to activities of daily living as measured by the MDS-UPDRS-II.

In addition to FOG itself, these results suggest that axial and lower limb parkinsonian signs associated with FOG are more severe in ONOFF-FOG, consistent with the interpretation that the overall presentation of ONOFF-FOG may have additional or separate pathophysiology. Visual inspection of graphics of MDS-UPDRS-III scores suggested that patients classified as ONOFF-FOG did not tend to cluster at, for example, higher OFF scores or lower amounts of MDS-UPDRS-III response; instead, they appeared across almost the total range of symptom levels and response levels (Fig. 1b, blue squares)

Other related MDS-UPDRS-III subdomains demonstrated limited impact of levodopa across the entire sample, including postural stability, speech, and leg agility, in which the most severe scores in the “ON” state were in the ONOFF-FOG group. Also, the most significant effects of group were identified for gait, toe tapping, and postural stability, with each being worse in the ONOFF-FOG group. No statistically significant state × group interactions were identified for MDS-UPDRS-III items other than FOG. It has been previously shown that FOG does not behave in a similar manner to bradykinesia.^13^ This suggests that FOG, sometimes referred to as the fifth cardinal feature,^14^ and related features behave differently from other cardinal features of PD in relation to levodopa responsiveness. These findings further support the notion that FOG, particularly in ONOFF-FOG cases, is governed by a separate pathophysiological mechanism than other cardinal features of PD.

Some suggest that FOG may progress through a continuum from responsive to unresponsive, indicating subtypes do not actually exist.^3^ Others indicate that ONOFF-FOG is an independent form that can come on without predating OFF-FOG.^4,9^ However, the histories of the individual patients in this study suggest that ONOFF-FOG can appear without first transitioning through OFF-FOG. The mean duration of FOG in the ONOFF-FOG group was 3.3 years as opposed to 4.4 years for the OFF-FOG group. If ONOFF-FOG was a later result of a cascade of changes then the duration would be expected to be longer. Further, we went back as far as 2006 in record review of all patients with freezing confirmed in the ON state in this cohort to examine if they previously exhibited OFF-FOG. Of these (*N* = 26 patients in total, including *N* = 19 classified as ONOFF-FOG and *N* = 7 with ON state freezing but subthreshold levodopa response), 10 patients had adequate data and clearly showed that they had ON state freezing since FOG inception. This is further support for ONOFF-FOG being a distinct entity as previously suggested.^8^ Additional studies with longitudinal follow-up will be necessary to confirm this.

We believe, based on these findings, that research studies of FOG should consider the levodopa responsiveness of FOG as an important clinical variable. In research projects, subjects are usually divided into those with and without FOG. This common approach may be therefore creating admixtures of ONOFF and OFF groups with high intra-group variability, creating conflicting results depending on what subtypes of FOG are more representative. One example of this involves the study of cognitive impairment in FOG. It is generally believed that FOG is associated with executive dysfunction and visuospatial changes and that this association is important from a pathophysiological standpoint.^15,16^ However, studies have actually demonstrated that these cognitive measures are associated specifically with ONOFF-FOG^10,17–19^ but not OFF-FOG.^10,20^ This approach could also explain why, for example, FOG severity has been associated with reduced functional connectivity within the ‘executive-attention’ neural network in the ‘resting’ state of some studies^21^ but not others.^22^

These findings also have important therapeutic implications. FOG observed in patients should be examined carefully to determine if they have OFF-FOG or ONOFF-FOG. One group, OFF-FOG, is treatable, responsive to levodopa and deep brain stimulation.^23^ The other, ONOFF-FOG is currently without treatment. A focus on the neurobiology of this particular problem could lead to the development of useful therapies. From the therapeutic standpoint of a physical or occupational therapist, it is well-established that FOG is a primary contributor to fall risk.^24^ Knowledge of whether FOG is levodopa-refractory could redirect therapeutic strategies to reduce fall risk from those focused on medical management to reduce OFF time to those focused on movement strategies to reduce fall risk during unavoidable FOG episodes that could occur at any time.

There are limits to this study. We used MDS-UPDRS-III item 11 to measure the presence of FOG in the “ON” and “OFF” states without further quantification of the severity of FOG. This was because our objective was just to examine persistence of FOG. Further study of severity will add more information to the levodopa response characteristics of FOG and therefore is a reasonable next step. We did not use blinded raters or randomize “ON” and “OFF” states or examination parts. Also, FOG tends to be variable in clinical presentation. It would be important to do a test-retest in such subjects to assure consistency of their clinical behavior before and after levodopa challenge. We did not perform longitudinal follow-up to examine change in responsiveness over time. Nevertheless, with careful examination and the use of levodopa blood levels we found consistency of levodopa response for cardinal features of PD from the “OFF” to the “ON” states and demonstrated the variance of levodopa responsiveness in relation to FOG. From a methodological perspective, we note that the medications used during the challenge dose were selected in a patient-specific manner based on the typical morning medication dose and the presence and severity of dyskinesia. This procedure was employed due to safety concerns. However, it has the potential to introduce unintended biases. Based on these findings, we now believe that a challenge dose including the typical morning dose of all medications with additional carbidopa/levodopa added to achieve 30% increase in levodopa equivalent vs. the typical morning dose would be safe, tolerable, and appropriate to introduce a full “ON” state in most patients.

We believe we have demonstrated the existence of ONOFF-FOG as a distinct subtype of FOG. In ONOFF-FOG there is also a diminished responsiveness of other axial features to levodopa. We also demonstrated that the impact of levodopa therapy on FOG varies from other cardinal features in PD. Further, ONOFF-FOG is not directly related to OFF-FOG. It would be an important next step to examine the neurobiology of ONOFF-FOG specifically and compare this to other levodopa responsive or induced subtypes.

## Methods

### Study population

This study was registered through clinicaltrials.gov (NCT02387281). Participants were recruited from the Emory Movement Disorders Clinic and provided written informed consent according to procedures approved by Emory University IRB. The study population included PD patients with and without FOG. This was based on question 1 of the New FOG questionnaire (NFOG-Q): “Did you experience “freezing episodes” over the past month?” However we also asked if they *ever* had FOG. Inclusion criteria were: Age ≥ 18 years; PD diagnosis according to United Kingdom Brain Bank criteria;^25^ Hoehn & Yahr stage I–IV in the OFF state; Demonstrated response to levodopa; able to sign a consent document and willing to participate in all aspects of the study. Additional inclusion criteria for participants with FOG were: FOG noted in medical history and confirmed visually by examiner. Exclusion criteria were: atypical parkinsonism including the presence of cerebrovascular disease or extensive white matter disease; prior treatment with medications that cause parkinsonism; neurological or orthopedic disorders interfering with gait; dementia or other medical problems precluding completion of study protocol.

### Clinical and demographic variables

Clinical and demographic data were collected using a battery of standardized instruments including the MDS-UPDRS Parts I, II, and IV. PD duration, FOG duration and ages at PD and FOG onset were taken via self-report. Self-reported FOG severity was assessed with the NFOG-Q.

### Levodopa challenge paradigm

Patients came to clinic in the practically defined “OFF” state >12 h after last intake of all antiparkinsonian medications. They were assessed for motor symptoms using the MDS-UPDRS-III. After the assessment phase, patients were administered a medication challenge dose that included all the medications typically taken in the morning (carbidopa/levodopa and other adjunctive agents), as well as additional carbidopa/levodopa administered in a patient-specific manner depending on whether or not dyskinesias were a known problem. Challenge medications were selected in order to achieve a levodopa equivalent dose (LED) of ~150% of the typical morning dose. However, dosage amounts were individualized to each patient by the examining investigator (SAF) based on whether dyskinesia was an issue and the size of their typical dose. For example, if a patient had a known moderate or worse dyskinetic response to their usual dose, a modest increased dose was given. If they had no or minor dyskinesia they were given a dose up to 200% of their usual dose. After administration of the challenge dose, patients were subsequently questioned at regular (~15 min) intervals until they indicated that they had reached their full “ON” state at which point the “ON” examination was completed (same measures as done in the “OFF” state). The interval between “OFF” and “ON” testing varied from 30 min to 3 h. Blood was drawn for measurement of levodopa level during the “OFF” state and immediately preceding the full “ON” assessment.

In order to increase the likelihood of producing FOG,^26^ the gait and balance testing protocols included a standard exam, timed-up-and go tests^27^ with and without a dual task,^28^ and rapid 360° turns.^26^ MDS-UPDRS-III score was used as a summary score for all these exams. Performance was scored in-person and scores were confirmed from video and amended if necessary (12/110 total scores). Identical testing procedures were used before and after the levodopa challenge dose.

We classified the response of each patient to the levodopa challenge as clinically-meaningful or not based on an observed improvement of ≥20% in MDS-UPDRS-III score after levodopa administration as utilized previously^29^ to minimize the number of patients with Pseudo-ON FOG and to ensure that patients were generally responsive to levodopa. Plots of improvement vs. MDS-UPDRS-III OFF score were examined for potential associations between improvement and baseline score or study group.

### Levodopa levels

Levodopa levels were performed to demonstrate that remaining FOG was not a consequence of inadequate levodopa dosage or delayed absorption and to assess appropriate response in relation to the level changes. A previously reported method was followed.^30^ HPLC with ESA 5600 A CoulArray electrochemical detection system, equipped with an ESA Model 584 pump and an ESA 542 refrigerated autosampler was used. Separations were performed at 35 °C using an MD-150 × 3.2 mm C18 column equipped with a C18 column guard cartridge. Levodopa levels were measured in ng/mg protein.

### FOG group assignment

The primary objective of the study was to demonstrate whether FOG persisted from the OFF to ON state. Hence we classified each patient into one of three study groups determined a priori based on history of the presence of FOG along with scores on the MDS-UPDRS-III item 11 “Freezing of Gait” exam in each of the “OFF” and “ON” medication states. Participants who had no history of FOG and received a score of zero on item 11 in both medication states were classified as “no freezing,” or “NO-FOG.” Participants who received a nonzero score on item 11 in the “OFF” medication state but a zero score in the “ON” medication state were classified as “OFF-FOG.” Participants who received a nonzero score on item 11 in both medication states were classified as “ONOFF-FOG.” All patients were categorized into study groups, independent of whether they exhibited a clinically meaningful response to the levodopa challenge test. Only patients that exhibited a clinically-meaningful response (≥20% improvement in MDS-UPDRS-III) to the levodopa challenge were included in analyses.

### Statistical analysis

We assessed differences in clinical and demographic characteristics across all three study groups with ANOVA and Tukey tests or chi-squared tests as appropriate. Comparisons of demographic and clinical variables specific to FOG (age at FOG onset, FOG duration, and NFOG-Q score) were performed only among the two FOG groups.

We used two approaches to analyze changes in parkinsonian symptom severity (MDS-UPDRS-III) and serum levodopa level between the “OFF” and “ON” medication state in each group. First, we used separate repeated measures ANOVAs to compare MDS-UPDRS-III scores and serum levodopa levels in each of the “OFF” and “ON” medication states across groups, with PD duration included as a covariate to control for imbalances across groups. MDS-UPDRS-III total scores were calculated without item III.11 “Freezing of Gait” as this item was used to establish group allocation. In these analyses, items with multiple subscores (e.g., item 16, kinetic tremor of the left and right upper limbs) were assembled into subscores prior to analysis.

Next, we used linear mixed models in SAS PROC MIXED to determine whether associations between MDS-UPDRS-III scores and serum levodopa level differed across the NO-FOG, OFF-FOG, or ONOFF-FOG groups. In these analyses, PD duration was included as a covariate and separate random intercepts and slopes were calculated for each patient. The OFF-FOG group was specified as the reference group to enable contrasts between the OFF-FOG and ONOFF-FOG groups. Satterthwaite’s approximation was used to estimate denominator degrees of freedom as necessary. Linear combinations of model coefficients were estimated with approximate *t*-tests.

Sample size was determined to achieve 80% power in discriminating performance on a cognitive outcome measure between groups. The results of those analyses will be reported separately. The sample size was determined to be sufficient based on a priori power in the overall study. Because the outcome measures in this study were not used to power the overall study, no adjustment was made for multiple outcomes. Each patient was treated as a distinct sample in primary analyses. In linear mixed models examining response to levodopa challenge, two measurements were made of each patient (OFF vs. ON) for each outcome measure. A repeated patient factor was included in these analyses to account for repeated measures. All summaries of central tendency are presented as mean ± standard deviation unless otherwise noted. All statistical tests were performed two sided at *ɑ* ≤ 0.05 in SAS University Edition release 3.8.

## Supplementary information

nr-reporting-summary

## Data Availability

The datasets generated during and/or analyzed during the current study are available from the corresponding author on reasonable request.
